# Does embryo culture at low oxygen tension improve ART outcomes?

**DOI:** 10.5935/1518-0557.20170001

**Published:** 2017

**Authors:** João Batista A Oliveira

**Affiliations:** 1 Center for Human Reproduction Prof Franco Jr

Even with the endogenous defense mechanisms present in oocytes and embryos, disturbances in embryonic culture processes may lead to an increased generation and buildup of reactive oxygen species (ROS) in culture media. This fact may be associated with various degrees of cellular damage (e.g. membrane disturbances, DNA fragmentation, etc.). Consequently, embryonic fragmentation, apoptosis, or even slowness or interruption of embryonic development is frequently seen. ROS may stem directly from gametes and embryos or in the environment in which they are found. The exposure of oocytes and embryos to xenobiotic agents, changes in metabolic substrate concentrations, and traces of transition elements favor the generation of ROS during *in vitro* culture/manipulation. High concentrations of oxygen is also listed as a harmful factor in this process.

Human and other mammalian embryos have traditionally been grown under atmospheric oxygen tension (~20%). However, experimental studies in several species of mammals have shown that the O_2_ concentration in the uterus and fallopian tubes generally fluctuates between 2-8%. Considering that such physiological hypoxia also exists in the human female genital tract, a reduction in oxygen levels could have important implications in laboratory practice. As with animal reports, beneficial effects of reduced levels of O_2_ were also detected in laboratory studies with humans, including a higher rate of embryonic development to the blastocyst stage, a faster cleavage rate, increased blastulation rate, Increase in the number of blastocysts and in the number of cryopreserved blastocysts, in addition to an increase in the ratio of high quality blastocysts. Regarding clinical outcomes, some studies have reported an increase in implantation rates, pregnancy rates, births and live births with low laboratory concentrations of O_2_ compared to its atmospheric concentrations. On the other hand, some randomized studies failed to obtain these positive results. As the results are conflicting, definitive conclusions to date cannot be drawn from individual studies.

However meta-analyzes on the subject did not show important evidence. A meta-analysis performed by our group (Gomes Sobrinho *et al*., 2011) concluded that, despite some promising results, additional randomized controlled trials are mandatory. A Cochrane Review (Bontekoe *et al*., 2012) suggests that culturing embryos under low oxygen concentrations improves the success rates of IVF and ICSI, resulting in the birth of more healthy newborns. However the methodological quality of the included trials was relatively low, and thus larger, multicenter clinical trials are needed to "get a better weighted overall view on the treatment effects of embryo culture under low oxygen concentrations in assisted reproductive technologies." A recent meta-analysis (Nastri *et al*., 2016) reaches the same conclusions 5 years after the first meta-analyzes, that despite small improvements with ~ 5% O_2_ in live birth/ongoing pregnancy and clinical pregnancy rates, the evidence is of very low quality and the best interpretation is that we are still very uncertain about differences found in this comparison.

Therefore, if the culture of human oocytes and embryos in low concentrations of oxygen can actually improve the clinical outcomes of assisted reproduction cycles, is a question that still requires more data. The current question is who would be willing to answer this question with randomized trials instead of new meta-analyses.
